# KRAS^G12C^ inhibitors versus chemotherapy alone for KRAS^G12C^-mutated non-small cell lung cancer: a pooled analysis of CodeBreaK 200 and KRYSTAL-12 trials

**DOI:** 10.3389/fonc.2026.1775677

**Published:** 2026-04-15

**Authors:** Shuang Liang, Yi Li, Ke Wan, Wenxiong Zhang, Linkai Xiong

**Affiliations:** 1 Department of Thoracic Oncology, Jiangxi Cancer Hospital & Institute (The Second Affiliated Hospital of Nanchang Medical College), Nanchang, China; 2 Nanchang Hangkong University Hospital, Nanchang, China; 3 Department of Thoracic Surgery, The Second Affiliated Hospital, Jiangxi Medical College, Nanchang University, Nanchang, China

**Keywords:** chemotherapy, KRAS, KRAS^G12C^ inhibitors, meta-analysis, non-small cell lung cancer

## Abstract

**Background:**

The KRAS^G12C^ mutation is a common oncogenic driver in non-small cell lung cancer (NSCLC). This study aimed to evaluate the clinical efficacy and safety profile of KRAS^G12C^ inhibitors (KGIs) compared with conventional chemotherapy regimens in patients with KRAS^G12C^-mutant NSCLC.

**Methods:**

A comprehensive literature search was undertaken across six databases to identify phase 3 randomized controlled trials (RCTs) evaluating KGIs versus conventional chemotherapy. Progression-free survival (PFS) was the primary endpoint, while secondary endpoints included central nervous system (CNS)-PFS, treatment responses, and treatment-related adverse events (TRAEs).

**Results:**

Two phase 3 RCTs (CodeBreaK 200 and KRYSTAL-12) involving 798 patients were included. The meta-analysis showed that KGIs significantly improved PFS (HR: 0.62 [0.51, 0.74], P < 0.00001) and CNS-PFS (HR: 0.55 [0.32, 0.94], P = 0.03). The survival rates of PFS at 1–12 months were also improved in the KGI group. Subgroup analyses demonstrated a consistent PFS benefit of KGIs over chemotherapy across all predefined subgroups. The objective response rate (ORR, RR: 2.73 [1.93, 3.85], P < 0.00001) and disease control rate (DCR, RR: 1.35 [1.22, 1.50], P < 0.00001) were also higher in the KGI group. Total and grade 3–5 TRAEs were similar between groups, although more TRAEs leading to dose interruption were observed in the KGI group (RR: 3.13 [2.37, 4.13], P < 0.00001). The top 3 grade 3–5 TRAEs in the KGI group were diarrhea (7.63%), alanine aminotransferase increased (7.63%), and aspartate aminotransferase increased (5.93%).

**Conclusion:**

KGIs demonstrated superior PFS, CNS-PFS, and response rates, with a manageable toxicity profile, suggesting that they represent a preferred treatment option for this population.

**Systematic Review Registration:**

https://www.crd.york.ac.uk/PROSPERO/view/CRD420251272701, identifier CRD420251272701.

## Introduction

Lung cancer accounts for the highest mortality burden among all cancers worldwide, and most diagnosed cases fall under the non-small cell lung cancer (NSCLC) category (1). The management of advanced NSCLC has been revolutionized by precision oncology, which directs treatment toward specific oncogenic drivers (2). Among these, KRAS mutations are highly prevalent, found in approximately 25-30% of lung adenocarcinomas (3). Historically, the KRAS protein was considered “undruggable” due to its structural features, leaving patients with this mutation with few targeted therapeutic options (4).

The KRAS^G12C^ mutation, a glycine-to-cysteine substitution, represents a critical and targetable subset, accounting for about 40% of all KRAS mutations in NSCLC (5). This mutation leads to constitutive activation of key downstream signaling pathways that promote tumor growth and survival (6). A pivotal therapeutic breakthrough was achieved with the development of covalent inhibitors that selectively bind to the mutant cysteine, such as sotorasib and adagrasib (7). In early-phase trials involving pretreated patients, these agents demonstrated substantial efficacy. Sotorasib showed an objective response rate (ORR) of 37.1% in the CodeBreaK 100 trial (8), while adagrasib achieved a 42.9% ORR in KRYSTAL-1 (9). These results led to accelerated regulatory approvals. However, definitive evidence from randomized comparisons against the previous standard-of-care, chemotherapy, was required to establish their comparative benefit.

This need has been met by two pivotal phase 3 randomized controlled trials (RCTs): CodeBreaK 200 (sotorasib vs. docetaxel) (10) and KRYSTAL-12 (adagrasib vs. docetaxel) (11). Although these individual trials provide high-level evidence, a meta-analysis synthesizing their data offers distinct advantages. It increases statistical power for more precise estimates of treatment effects and safety outcomes, allows for a unified evaluation of the drug class, and strengthens the evidence base for clinical guidelines. Therefore, this study systematically pooled data from the two phase 3 RCTs to assess the clinical benefits and risk profile of KRAS^G12C^ inhibitors (KGIs) versus conventional chemotherapy in patients with KRAS^G12C^-mutant NSCLC.

## Materials and methods

### Search strategy

Our comprehensive literature search employed the keywords “KRAS”, “Lung cancer”, and “Randomized”. We queried six databases (PubMed, ScienceDirect, Cochrane Library, Scopus, EMBASE, and Web of Science), covering all records available up to November 24, 2025 (**Supplementary Table S1**). In addition to published literature, relevant clinical trial registries (e.g., ClinicalTrials.gov, EU Clinical Trials Register) were searched to identify any unpublished studies.

### Selection criteria

Inclusion criteria:

1. Participants (P): Individuals diagnosed with NSCLC harboring a KRAS^G12C^ mutation;
2. Intervention (I) and Control (C): KGIs and standard chemotherapy regimens;
3. Outcomes (O): Progression-free survival (PFS) and overall survival (OS) were defined as co-primary endpoints. Additional measures encompassed central nervous system (CNS)-PFS, tumor response metrics, and safety profiles regarding treatment-related adverse events (TRAEs).
4. Study design (S): RCTs.

Exclusion criteria: (1) retrospective study, abstracts/conference reports, single arm study, case report, review, or meta-analysis; (2) animal studies; (3) studies that lacked adequately reported or analytically suitable data.

### Data extraction

Two researchers gathered information on study characteristics (trial name, registration number, etc.), patient attributes (count, gender, etc.), survival results (PFS, CNS-PFS, etc.), response metrics (ORR, complete response [CR], etc.), and TRAEs. Missing information was asked from the corresponding authors, and conflicts were resolved through re-assessment.

### Outcome assessments

PFS was evaluated across predefined strata, including age, sex, geographic region, ECOG PS, smoking status, histological type, disease stage, metastases (quantity and location), and PD-L1 expression. A landmark approach was applied to assess PFSR from month 1 through month 12 and CNS-PFSR over the same intervals between treatment arms. Kaplan–Meier plots served as the source for survival estimates, which were digitized using Engauge Digitizer version 12.1. For survival rates, risk ratio (RR) and 95% confidence intervals (CIs) were derived using the Mantel–Haenszel technique. Differences in survival proportions between the KGI arm and the chemotherapy arm at each time landmark were examined through chi-square testing.

### Quality assessment

Evaluation of trial rigor was conducted by applying the Cochrane Risk Assessment Tool in combination with the Jadad scoring method, allowing a maximum score of seven points, with scores between five and seven reflecting strong methodological quality (12, 13). Evidence certainty was then appraised through the GRADE framework, which stratifies findings into four hierarchical categories, ranging from very low to high confidence (14).

### Statistical analysis

Quantitative synthesis was carried out using Review Manager (v5.3) and STATA (v12.0). In cases where trials released multiple datasets over time, analyses preferentially incorporated the most recent information. When updated reports omitted certain endpoints, corresponding values were retrieved from earlier publications. Associations involving survival variables were summarized by HR, whereas dichotomous variables were expressed as RR. Heterogeneity among studies was assessed using the *I²* statistic and Cochran’s Q test. Choice of analytical framework was guided by inconsistency magnitude, applying fixed-effects model under limited heterogeneity (I² < 50% or P > 0.1) and switching to a random-effects model as heterogeneity was substantial. A P value below 0.05 was interpreted as evidence of statistical significance. Publication bias was assessed through graphical evaluation of funnel plot asymmetry.

## Results

### Search results

**Figure 1:**
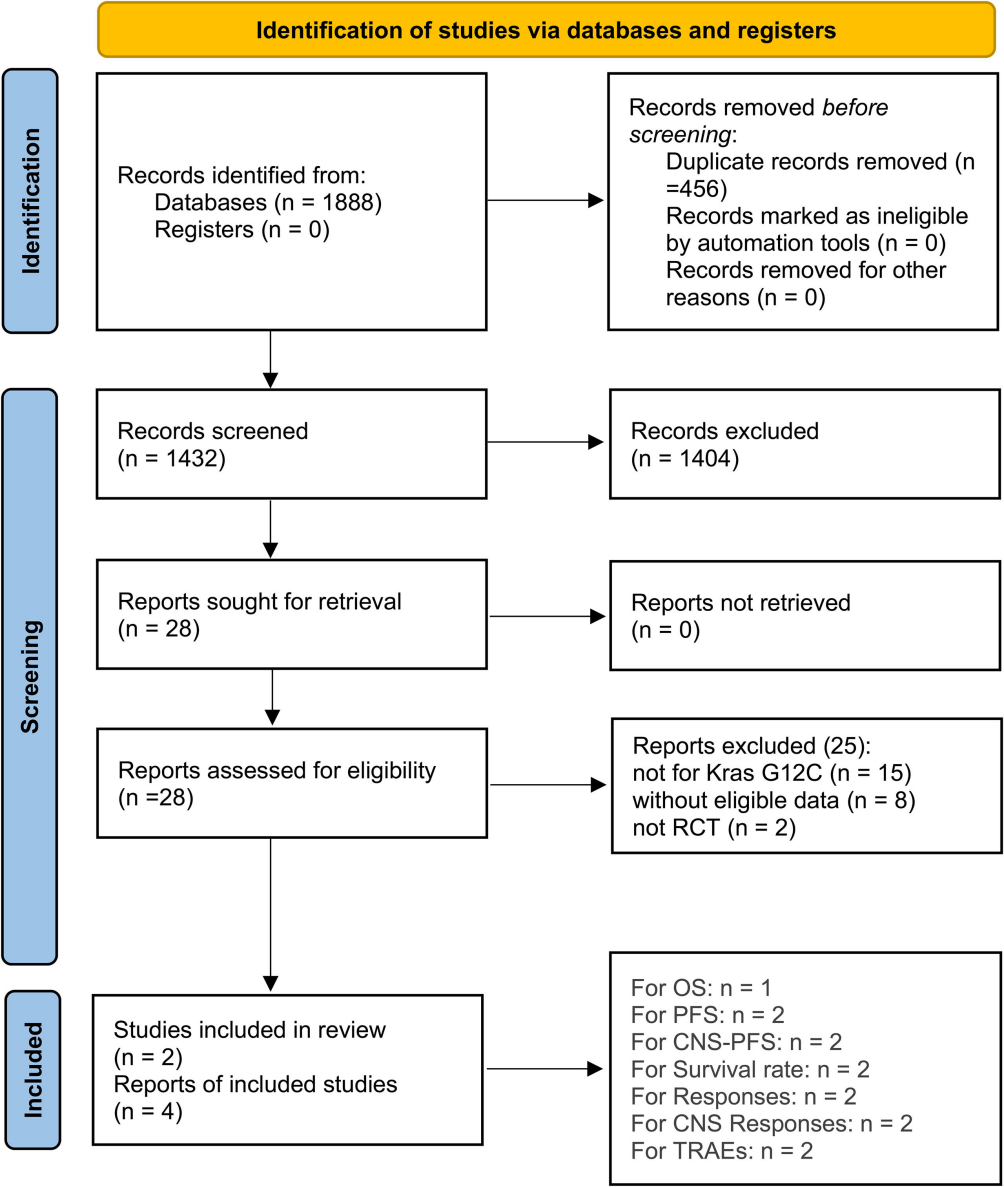
Study selection and inclusion process.

A total of 1432 studies were initially identified, after which four eligible articles corresponding to two phase III RCTs—CodeBreaK 200 and KRYSTAL-12—were selected, collectively involving 798 individuals (**Figure 1**) (10, 11, 15, 16). These studies were conducted as multinational, multicenter investigations, and methodological appraisal rated both trials at a high-quality level (**Supplementary Figure S1**; **Supplementary Table S2**). Participant demographics and clinical baseline data are presented in **Table 1**. Using the GRADE framework, overall evidence certainty for reported outcomes was judged to fall within the medium-to-high range (**Supplementary Table S3**).

**Table 1:** Baseline features of the CodeBreaK 200 and KRYSTAL-12 trials.

Characteristics	CodeBreaK 200		KRYSTAL-12	
Register number	NCT04303780		NCT04685135	
Design	RCT		RCT	
Clinical trial stage	Phase 3		Phase 3	
Included articles	Dingemans 2025 (15), Waterhouse 2024 (16), de Langen 2023 (10)		Barlesi 2025 (11)	
Country	Global Multicenter		Global Multicenter	
Period	2020.06-2021.04		2021.02-2023.11	
Treatment arms	KGI	Chemotherapy	KGI	Chemotherapy
Therapy	Sotorasib	Docetaxel	Adagrasib	Docetaxel
Patients (n)	171	174	301	152
Sex (M/F)	109/62	95/79	193/108	110/42
Median age (year)	64	64	64	65
Smoking status				
Former or current	166	166	284	143
Never	5	8	17	9
ECOG PS				
0	59	59	96	47
1	112	115	205	104
Race				
White	142	144	135	81
Asian	21	22	72	37
Others	8	8	94	34
Histology				
Squamous	1	7	6	0
Adenocarcinoma	169	165	283	147
Others	1	2	12	5
Stage				
III	9	8	18	8
IV	162	166	283	144
Baseline metastases				
Brain	58	60	78	36
Liver	30	35	46	18
Bone	81	69	68	39
PD-L1 expression				
<1%	57	55	61	34
1-49%	46	70	126	69
>50%	60	40	71	29
Previous lines of therapy in advanced or metastatic setting				
1	77	78	207	110
2	65	69	74	35
2+	29	27	20	7
Follow-up duration (months)	20	20	7.2	7.2

ECOG PS, Eastern cooperative oncology group performance status; KGI, KRASG12C inhibitor; M/F, Male/Female; PD-L1, Programmed death-ligand 1; RCT, Randomized controlled trial.

### Survival

**Figure 2:**
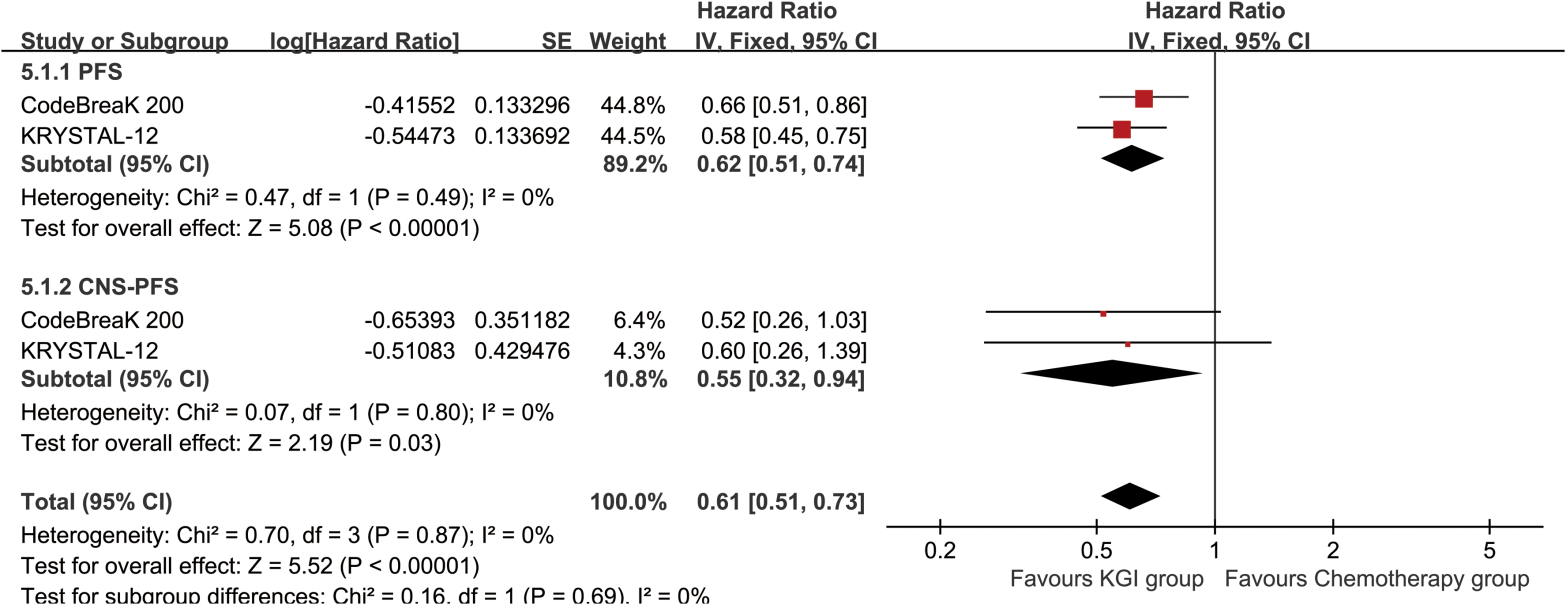
Forest plots depicting PFS and CNS-PFS for KGIs compared with chemotherapy.

**Figure 3:**
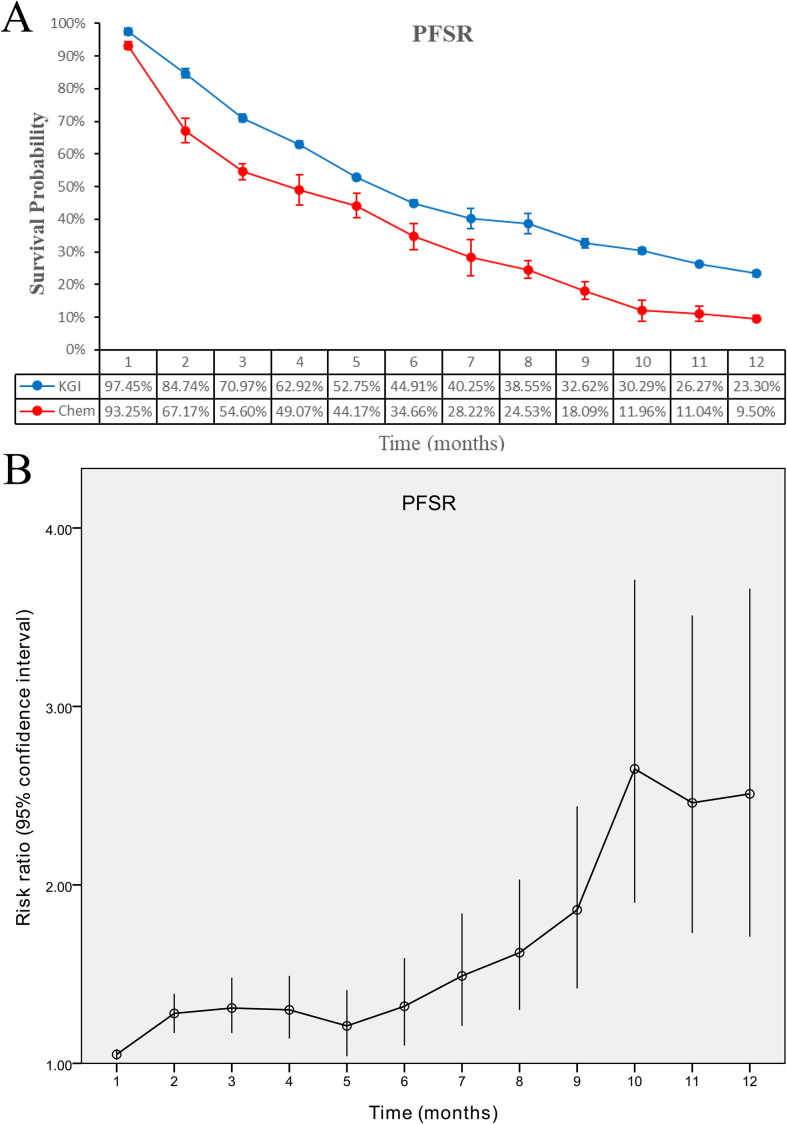
Comparative analysis of PFSR for KGIs versus chemotherapy. **(A)** PFSR over 1–12 months; **(B)** corresponding risk ratios.

Compared with the chemotherapy arm, treatment with KGIs was associated with a prolonged PFS, with an HR of 0.62 (95% CI: 0.51-0.74, P < 0.00001) (**Figure 2**). In addition, patients receiving KGIs exhibited consistently elevated PFSR across all evaluated time points from month 1 through month 12 (**Figure 3**; **Supplementary Figure S2**).

**Figure 4:**
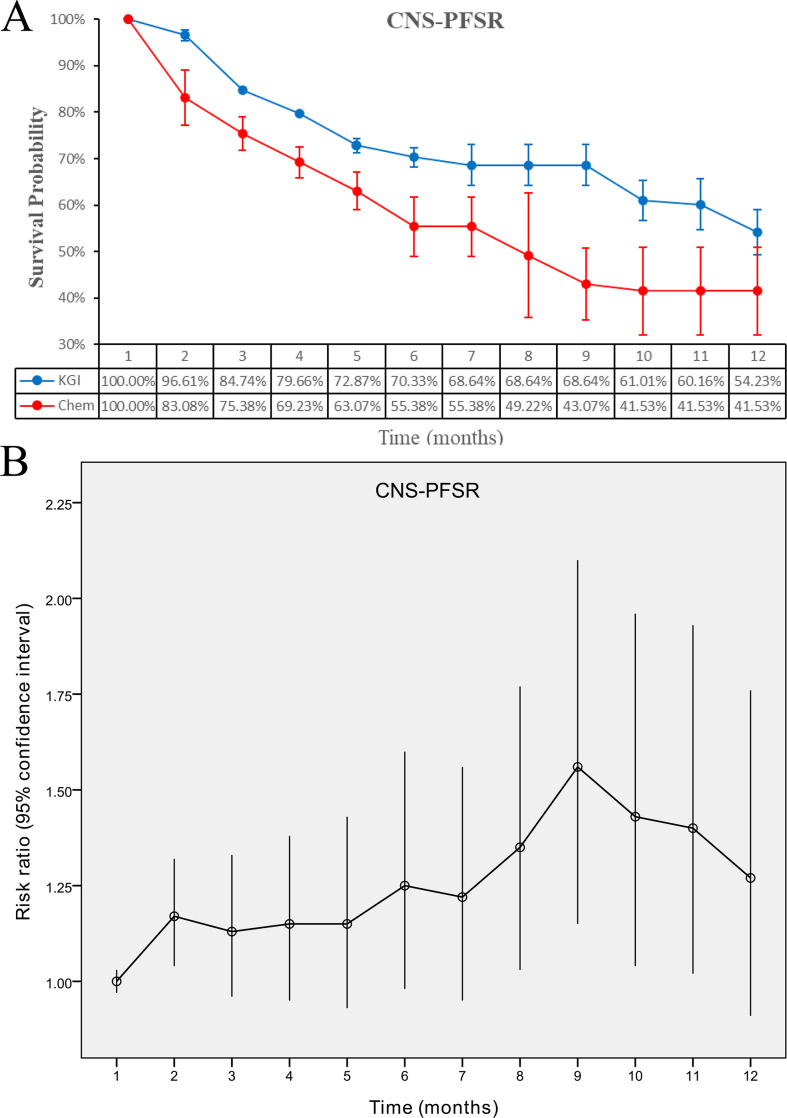
Comparative analysis of CNS-PFSR for KGIs versus chemotherapy. **(A)** CNS-PFSR over 1–12 months; **(B)** corresponding risk ratios.

Meanwhile, patients receiving KGIs demonstrated a favorable CNS-PFS, reflected by an HR of 0.55 (95% CI: 0.32-0.94, P = 0.03) (**Figure 2**). In parallel, CNS-PFSR values were consistently greater in the KGI arm at months 2 and 9-11 (**Figure 4**; **Supplementary Figure S3**).

Only CodeBreaK 200 reported the data of overall survival (OS) and showed similar OS between the two groups (HR: 1.01 [0.77, 1.33], P = 0.53) (10).

### Subgroup analysis of survival

According to predefined subgroups (detailed in the outcome assessments), PFS benefit of KGIs over chemotherapy was observed across all subgroups. The presence of liver metastases appears to favor KRAS G12C inhibitor therapy (HR: 0.45 [0.29, 0.70], P = 0.0004) (**Table 2**).

**Table 2:** Subgroup analyses of progression-free survival.

Subgroups	Involved studies	Progression-free survival			
		Patients	HR (95% CI)	*I^2^*	*P*
All patients	2	798	0.62 [0.51, 0.74]	0	< 0.00001
Age					
< 65 years	2	420	0.62 [0.48, 0.79]	0	< 0.0001
> 65 years	2	378	0.62 [0.46, 0.83]	0	0.001
Sex					
Female	2	291	0.67 [0.48, 0.93]	0	0.02
Male	2	507	0.55 [0.44, 0.70]	0	< 0.00001
Geographic region					
Asia	2	161	0.56 [0.36, 0.86]	45%	0.008
Non-Asia	2	635	0.63 [0.51, 0.78]	30%	< 0.0001
ECOG PS					
0	2	261	0.53 [0.37, 0.76]	0	0.0006
1	2	536	0.61 [0.49, 0.76]	0	< 0.0001
Smoking status					
Current/former	2	758	0.61 [0.50, 0.75]	0	< 0.00001
Never	1	26	0.70 [0.26, 1.88]	–	0.48
Brain metastases					
Yes	2	232	0.60 [0.43, 0.83]	0	0.002
No	2	566	0.62 [0.50, 0.78]	47%	< 0.0001
Liver metastases					
Yes	2	129	0.45 [0.29, 0.70]	0	0.0004
No	2	669	0.62 [0.51, 0.77]	0	< 0.00001
Bone metastases					
Yes	2	257	0.61 [0.45, 0.82]	0	0.001
No	2	541	0.58 [0.46, 0.74]	0	< 0.00001
PD-L1 expression					
<1%	2	207	0.56 [0.39, 0.81]	11%	0.002
1%-49%	2	311	0.58 [0.43, 0.78]	0	0.0003
>50%	2	200	0.68 [0.46, 1.01]	0	0.06

CI, Confidence interval; ECOG PS, Eastern cooperative oncology group performance status; HR, Hazard ratio; I², I-squared statistic; P, Probability; PD-L1, Programmed death-ligand 1.

### Responses

**Figure 5:**
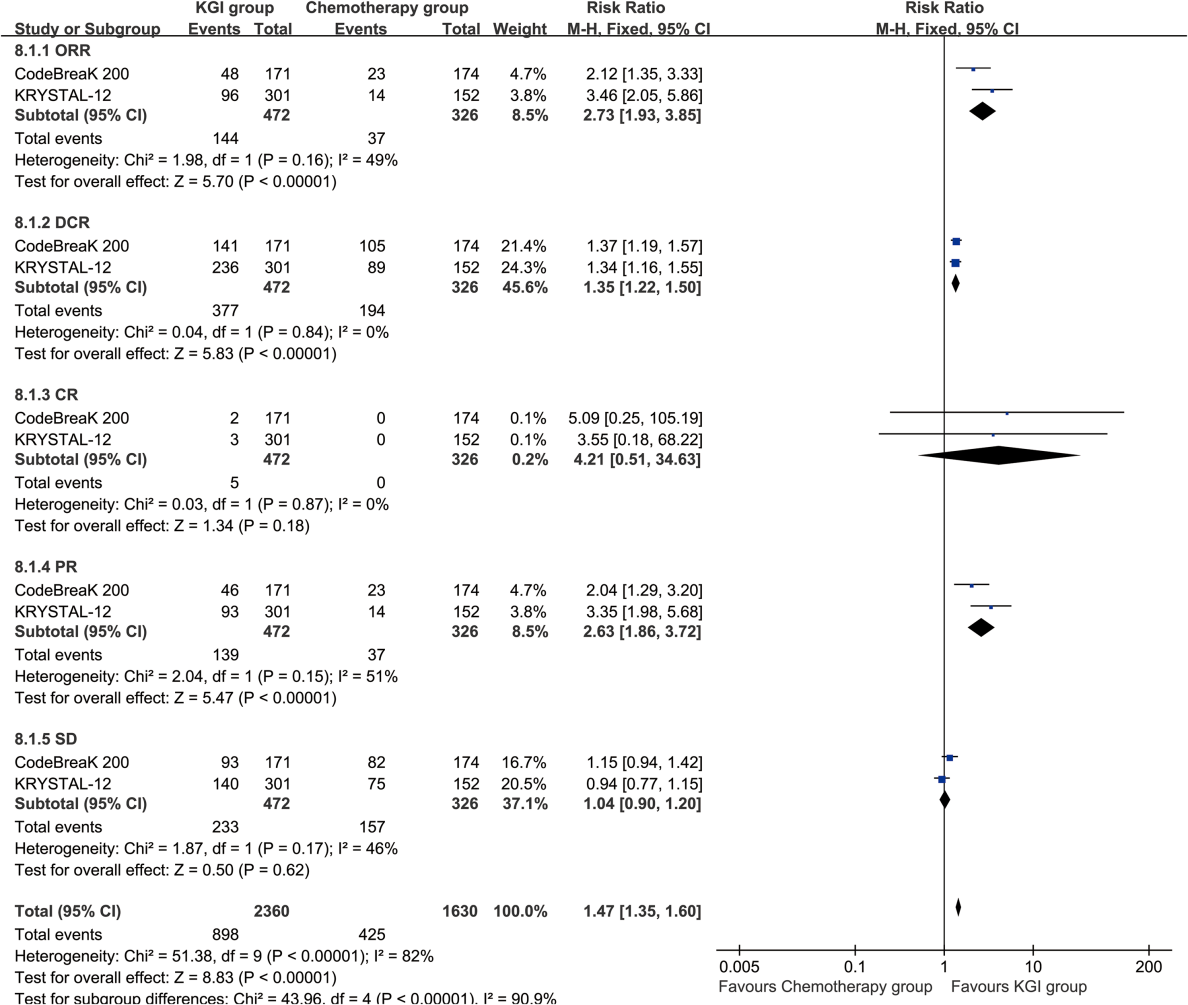
Forest plots depicting overall response rates for KGIs compared with chemotherapy.

In the analysis of overall response, the ORR (RR: 2.73 [1.93, 3.85], P < 0.00001), disease control rate (DCR, RR: 1.35 [1.22, 1.50], P < 0.00001), and partial response (PR, RR: 2.63 [1.86, 3.72], P < 0.00001) were higher in the KGI group. The progressive disease (PD, RR: 0.49 [0.39, 0.61], P < 0.00001) was less in the KGI group (**Table 3**; **Figure 5**).

**Table 3:** Overall and CNS response outcomes.

Responses	KGI		Chemotherapy		Risk ratio [95% CI]	*I^2^*	P
	Event/total	%	Event/total	%			
Overall response							
ORR	144/472	30.51%	37/326	11.35%	2.73 [1.93, 3.85]	49%	< 0.00001
DCR	377/472	79.87%	194/326	59.51%	1.35 [1.22, 1.50]	0	< 0.00001
CR	5/472	1.06%	0/326	0.00%	4.21 [0.51, 34.63]	0	0.18
PR	139/472	29.45%	37/326	11.35%	2.63 [1.86, 3.72]	51%	< 0.00001
SD	233/472	49.36%	157/326	48.16%	1.04 [0.90, 1.20]	46%	0.62
PD	95/472	20.13%	132/326	40.49%	0.49 [0.39, 0.61]	0	< 0.00001
CNS response							
CNS-ORR	25/96	26.04%	6/49	12.24%	2.18 [0.96, 4.98]	0	0.06
CNS-DCR	79/96	82.29%	31/49	63.27%	1.21 [0.78, 1.86]	75%	0.40
CNS-CR	12/96	12.50%	4/49	8.16%	1.48 [0.50, 4.41]	0	0.48
CNS-PR	13/96	13.54%	2/49	4.08%	3.66 [0.86, 15.49]	0	0.08
CNS-SD	54/96	56.25%	25/49	51.02%	1.11 [0.79, 1.56]	62%	0.54
CNS-PD	17/96	17.71%	18/49	36.73%	0.47 [0.27, 0.82]	19%	0.008

CNS, Central nervous system; CR, Complete response; CI, confidence interval; I², I-squared statistic; KGI, KRASG12C inhibitor; DCR, Disease control rate; ORR, Objective response rate; P, Probability; PD, Progressive disease; PR, Partial response; RR, Risk ratio; SD, Stable disease.

In the analysis of CNS response, the CNS-PD (RR: 0.47 [0.27, 0.82], P = 0.008) was also less in the KGI group. The CNS-ORR, CNS-DCR, CNS-CR, and CNS-PR tended to favor the KGI group without statistical significance (**Table 3**; **Supplementary Figure S4**).

### Safety

Overall, treatment with KGI was associated with a markedly increased frequency of TRAEs leading to dose interruption (RR: 3.13 [2.37, 4.13], P < 0.00001). By contrast, no significant differences were observed between study arms in terms of overall/grade 3-5/serious TRAEs, and TRAEs leading to dose reduction/discontinuation/deaths (**Table 4**; **Supplementary Figure S5**).

**Table 4:** Summary of treatment-related adverse events.

TRAEs	KGI		Chemotherapy		Risk ratio [95% CI]	*I^2^*	P
	Event/total	%	Event/total	%			
Total TRAEs	399/472	84.53%	251/326	76.99%	1.05 [0.83, 1.32]	88%	0.68
Grade 3–5 TRAEs	196/472	41.53%	125/326	38.34%	1.03 [0.87, 1.23]	0	0.71
Serious TRAEs	80/472	16.95%	57/326	17.48%	0.87 [0.35, 2.15]	86%	0.76
TRAEs leading to dose reduction	169/472	35.81%	73/326	22.39%	1.22 [0.38, 3.93]	94%	0.74
TRAEs leading to dose interruption	237/472	50.21%	49/326	15.03%	3.13 [2.37, 4.13]	0	< 0.00001
TRAEs leading to discontinuation	39/472	8.26%	37/326	11.35%	0.73 [0.45, 1.18]	23%	0.20
TRAEs leading to deaths	5/472	1.06%	3/326	0.92%	1.12 [0.24, 5.12]	0	0.89

CI, Confidence interval; I², I-squared statistic; KGI, KRASG12C inhibitor; P, Probability; RR, Risk ratio; TRAE, Treatment-related adverse event.

In the analysis of any grade TRAEs, more diarrhea, alanine aminotransferase (ALT) increased, aspartate aminotransferase (AST) increased, blood creatinine increased, γ-Glutamyltransferase increased, blood alkaline phosphatase (ALP) increased, and lipase increased were found in the KGI group. Conversely, more asthenia, white blood cell count decreased, neutrophil count decreased, constipation, dysgeusia, neutropenia, myalgia, arthralgia, alopecia, mucositis, neuropathy peripheral, and oedema peripheral were found in the chemotherapy group (**Supplementary Table S4**).

In the analysis of grade 3–5 TRAEs, more ALT increased, AST increased, and blood ALP increased were found in the KGI group. Conversely, more asthenia, white blood cell count decreased, neutrophil count decreased, and neutropenia were found in the chemotherapy group (**Supplementary Table S5**).

### Publication bias

**Figure 6:**
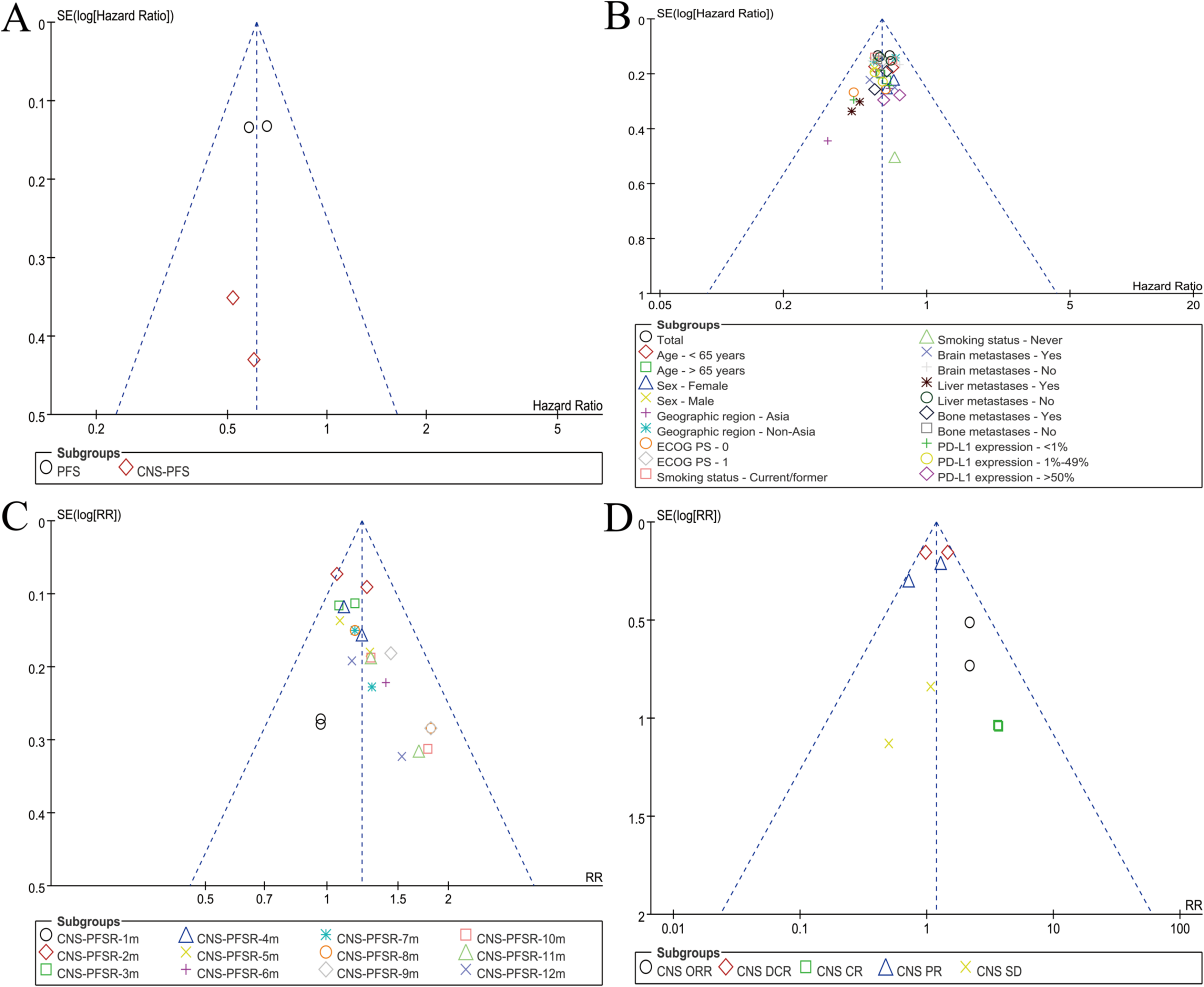
Funnel plots assessing publication bias: **(A)** survival, **(B)** subgroup analysis of PFS, **(C)** CNS-PFSR, and **(D)** CNS response rates.

Visual inspection of the funnel plot for survival outcomes, PFS subgroup analyses, CNS-PFSR, and CNS response rates indicated that potential publication bias remained within an acceptable range (**Figure 6**).

## Discussion

The therapeutic landscape for advanced NSCLC with KRAS^G12C^ mutations has changed dramatically with the advent of direct inhibitors. For years, the “undruggable” nature of KRAS limited patients to chemotherapy or immunotherapy, which yielded suboptimal and unpredictable outcomes, highlighting a significant unmet need (17). The accelerated approvals of sotorasib and adagrasib were landmark events, yet they were based on single-arm studies (7, 8). Although these studies demonstrated clear activity, they left critical questions regarding the magnitude of benefit relative to the previous standard, docetaxel chemotherapy, unanswered. The subsequent publication of the CodeBreaK 200 and KRYSTAL-12 trials provided the necessary randomized evidence, and a synthesized, quantitative summary is needed to improve precision and evaluate the drug class as a whole for clinicians and guideline committees (10, 11). Our pooled analysis directly addresses this need, providing the highest level of evidence synthesis for this defined population. By integrating data from these two pivotal phase 3 RCTs, our analysis robustly confirms that KGIs provide statistically significant improvements in PFS and CNS-PFS compared with chemotherapy, as well as superior ORR and DCR. The safety profile, though distinct and associated with more dose interruptions, remains manageable and differs in spectrum from chemotherapy. These findings solidify the position of KGIs as the preferred standard of care for previously treated patients with KRAS^G12C^-mutant advanced NSCLC.

The most compelling finding of this analysis is the consistent and substantial PFS benefit, with a pooled HR of 0.62, corresponding to a 38% reduction in the risk of progression, representing a major therapeutic advance. Notably, our landmark analysis further delineates this benefit, demonstrating superior PFS rates in the KGI group from as early as 1 month and sustained through 12 months. Subgroup analyses reinforced the robustness of this PFS advantage, showing benefit across all predefined patient categories, including patients with poor prognostic features such as brain or liver metastases. Interestingly, patients with liver metastases derived an even greater PFS benefit (HR: 0.45), a finding that warrants further investigation and may relate to the pharmacokinetics or specific biology of hepatic disease. The significant improvement in CNS-PFS (HR: 0.55) is a particularly critical outcome. Brain metastases are a frequent and devastating complication in KRAS-mutant NSCLC, associated with poor quality of life and survival (18). Traditional chemotherapy has limited CNS penetration (19). This represents a distinct advantage over chemotherapy, adding an important clinical benefit by potentially delaying the need for local therapies such as radiotherapy. The lack of a statistically significant OS benefit, based on mature data from CodeBreaK 200, presents a crucial point for discussion—a so-called ‘PFS–OS dissociation’ (10). This phenomenon is not uncommon in oncology trials, particularly when effective subsequent therapies are available (20). In both trials, a substantial proportion of patients in the docetaxel arm crossed over to receive a KGI upon progression, which could confound OS analysis by diluting potential survival differences between the randomized groups (8, 10). Furthermore, the availability of other later-line options, including immunotherapy combinations, may also impact OS. Importantly, OS remains immature for KRYSTAL-12. It is also biologically plausible that while KGIs effectively suppress tumor growth initially, the development of diverse resistance mechanisms—such as secondary KRAS mutations, bypass pathway activation (e.g., via RTKs, RAS, or RAF), or histologic transformation—limits their ability to confer a long-term survival advantage in all patients (21–23). Therefore, the current OS data should not diminish the clear clinical benefit of superior PFS and response rates, which translate into delayed disease progression, extended time free from chemotherapy toxicities, and improved symptom control. These observations highlight potential differences in efficacy profiles between Sotorasib and Adagrasib, particularly regarding CNS activity, although definitive conclusions require direct comparative studies. This underscores the importance of considering drug-specific characteristics when making clinical treatment decisions.

Our analysis confirms that the PFS benefit is underpinned by superior tumor shrinkage and control. KGIs nearly tripled the ORR (RR: 2.73) and significantly improved DCR (RR: 1.35), highlighting their clear efficacy. Partial response (PR) was the predominant response type, consistent with cytostatic growth inhibition rather than cytotoxic tumor lysis. The lower rate of PD as best response in the KGI arm further emphasizes that these agents provide meaningful disease stabilization for most patients, an important outcome in the late-line setting (24). Although CNS-specific ORR, CR, and PR did not reach statistical significance, the data showed a strong trend favoring KGIs and a significant reduction in CNS progression as the best response. These findings align with CNS-PFS results and are supported by biomarker analyses from early-phase trials demonstrating drug penetration and target engagement in cerebrospinal fluid (25, 26). Challenges in assessing CNS responses, particularly in often asymptomatic or previously treated brain lesions, may contribute to the lack of statistical significance for classical response metrics (27). Nonetheless, the collective evidence on CNS outcomes strongly supports KGI activity in this sanctuary site.

The safety analysis highlights a fundamentally different toxicity profile between targeted therapy and chemotherapy. While overall incidences of any-grade and severe TRAEs were comparable between arms, the incidence of individual TRAEs varied considerably. The KGI safety signature is dominated by hepatotoxicity (elevated ALT, AST, ALP) and gastrointestinal effects (diarrhea), which are on-target and manageable with monitoring, dose adjustments, and supportive care per current guidelines (28, 29). This is reflected in our finding of a three-fold higher rate of dose interruptions in the KGI group, a proactive strategy to manage these reversible toxicities without requiring permanent discontinuation in most patients. In contrast, the chemotherapy profile was characterized by hematological toxicities (neutropenia, leukopenia), constitutional symptoms (asthenia), and neuro-sensory effects (neuropathy, dysgeusia)—toxicities that significantly impact patient quality of life and pose risks of serious infection (29). The similar rates of serious TRAEs, discontinuations, and fatal events between arms indicate that the novel toxicities of KGIs, while requiring different management, do not constitute an unacceptable safety risk. This favorable risk-benefit ratio is central to their clinical adoption. Emerging research suggests that KRAS^G12C^ inhibition may have immunomodulatory effects, potentially influencing the safety and efficacy of future immunotherapy combinations (30).

Our analysis has several limitations. First, this study is a study-level meta-analysis of only two RCTs; although high-quality, this limits exploration of heterogeneity and precludes patient-level analyses that could identify more precise predictive biomarkers. Second, OS data, particularly for adagrasib, are not yet fully mature and will require future updates. Third, this analysis focuses on the second-line setting; the evolving role of KGIs in the first-line, either as monotherapy or in combination with other agents, was not addressed and remains under active investigation (31). Furthermore, not all patients with KRAS^G12C^ mutations respond to KGIs, and acquired resistance is nearly universal. This analysis cannot address the critical need for predictive biomarkers for primary resistance.

## Conclusion

In summary, KGIs demonstrate superior efficacy over chemotherapy in patients with KRAS^G12C^-mutant advanced NSCLC, significantly improving PFS, CNS-PFS, and tumor response rates. The PFS benefit of KGIs was consistent across all predefined subgroups. Their distinct yet manageable safety profile supports a favorable therapeutic index. These findings firmly establish KGIs as a standard-of-care option for this patient population. Future efforts should focus on elucidating resistance mechanisms, developing effective combination strategies, and optimally integrating these agents into the broader treatment sequence, including first-line immunotherapy.

## Data Availability

The data presented in the study are derived from publicly available published articles, including the CodeBreaK 200 and KRYSTAL-12 trials, and can be accessed through the respective journal websites. The sources are available at: CodeBreaK 200: https://www.thelancet.com/journals/lancet/article/PIIS0140-6736(23)00221-0/abstractKRYSTAL-12: https://www.thelancet.com/journals/lancet/article/PIIS0140-6736(25)00866-9/abstract.

## Supplementary material

Supplementary Figure S1 Assessment of risk of bias using the Cochrane tool.

Supplementary Figure S2 Forest plots depicting PFSR at 1–12 months for KGIs compared with chemotherapy.

Supplementary Figure S3 Forest plots depicting CNS-PFSR at 1–12 months for KGIs compared with chemotherapy.

Supplementary Figure S4 Forest plots depicting CNS response rates for KGIs compared with chemotherapy.

Supplementary Figure S5 Forest plots depicting safety summary for KGIs compared with chemotherapy.

Supplementary Table S1 Detailed literature search strategy.

Supplementary Table S2 Methodological quality of included studies assessed by the Jadad scale.

Supplementary Table S3 GRADE evaluation of evidence by treatment modality and study design.

Supplementary Table S4 Any grade treatment-related adverse events.

Supplementary Table S5 Grade 3–5 treatment-related adverse events.
