# Supplementary figures and images for: KRASG12C inhibitors versus chemotherapy alone for KRASG12C-mutated non-small cell lung cancer: a pooled analysis of CodeBreaK 200 and KRYSTAL-12 trials
Source: Front Oncol. 2026 Apr 15;16:1775677. doi: 10.3389/fonc.2026.1775677 (PMC13124570; doi:10.3389/fonc.2026.1775677)

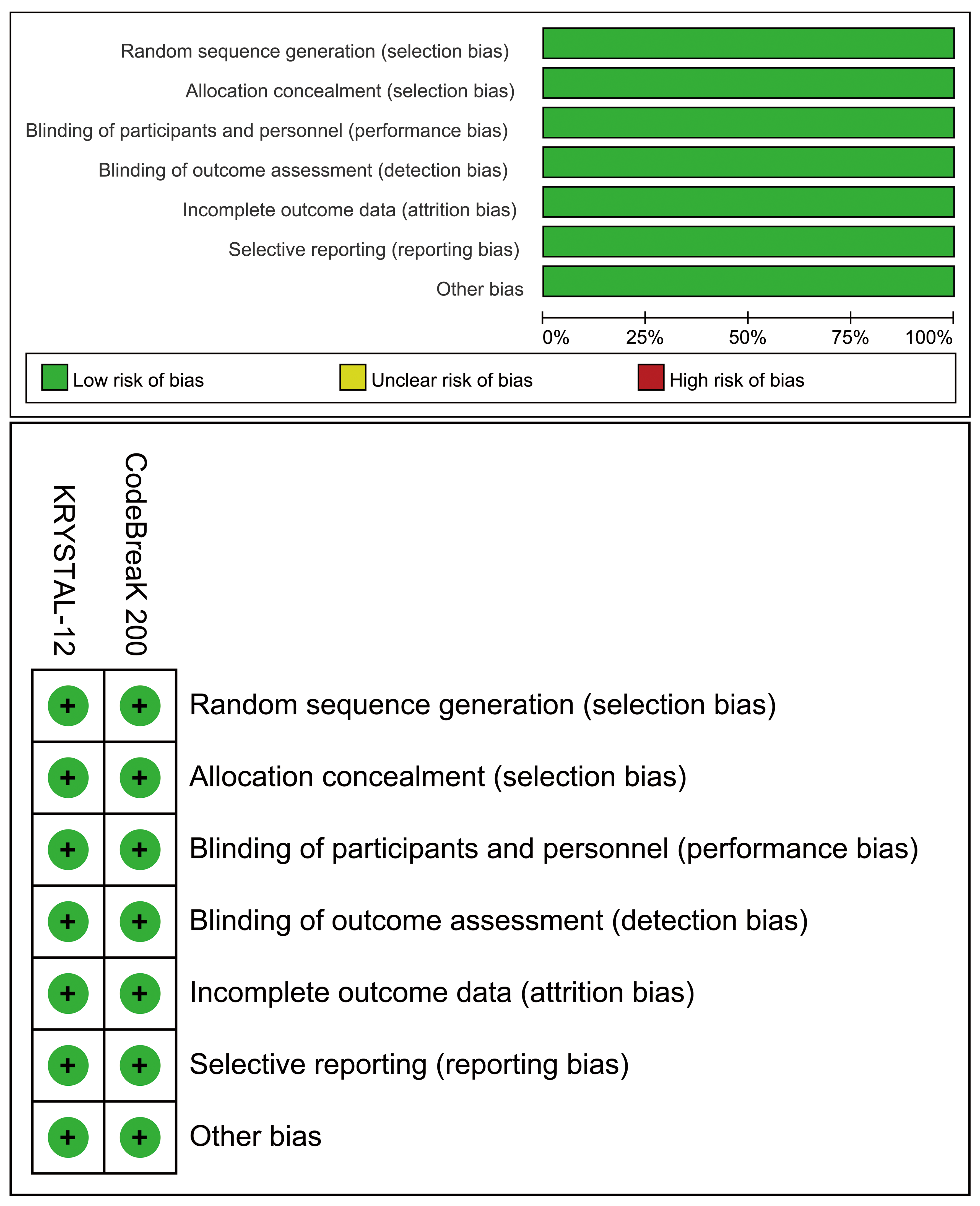

Supplement: Supplementary Figure S1 — Assessment of risk of bias using the Cochrane tool. [file Image1.tif]

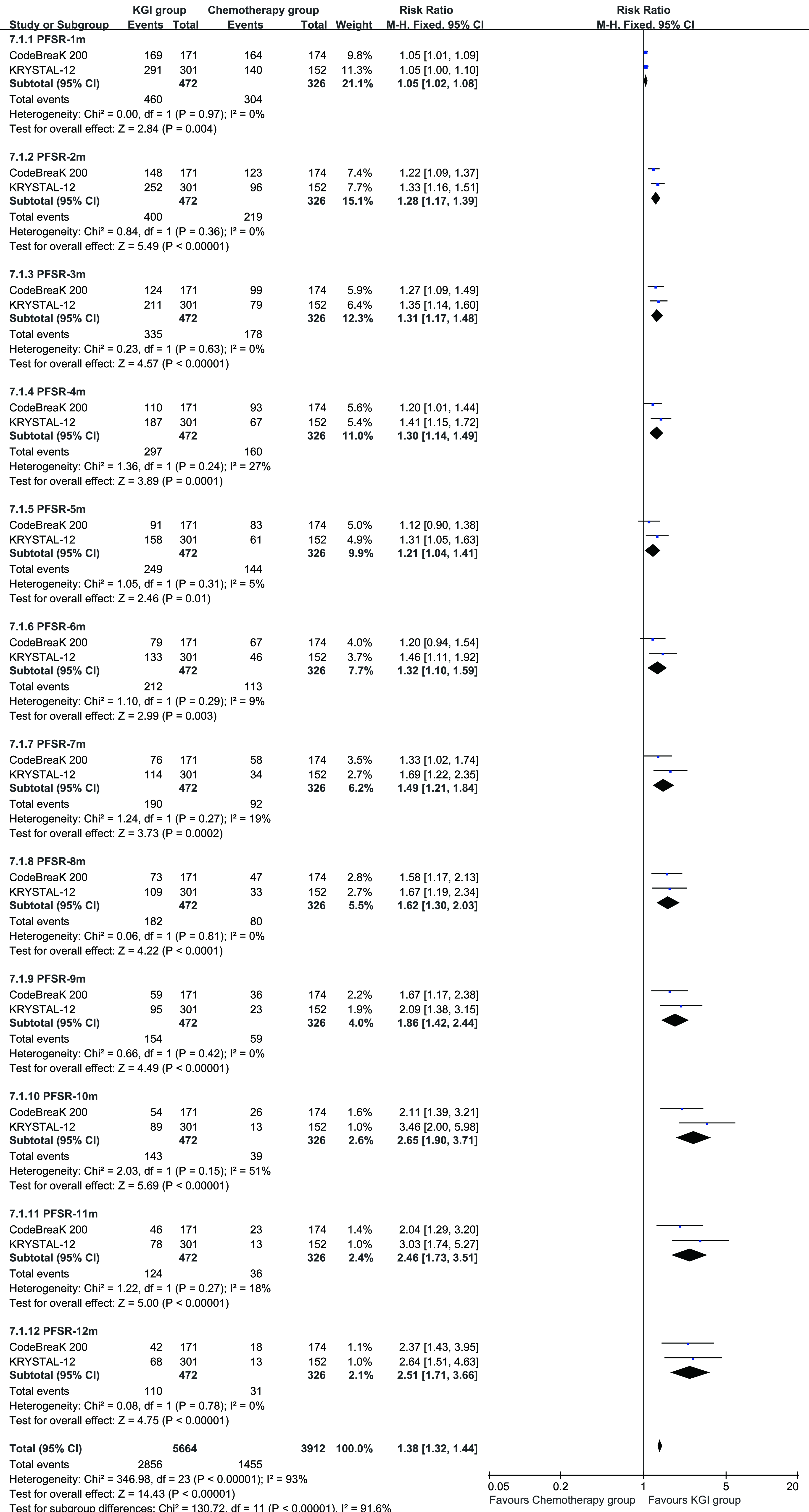

Supplement: Supplementary Figure S2 — Forest plots depicting PFSR at 1–12 months for KGIs compared with chemotherapy. [file Image2.tif]

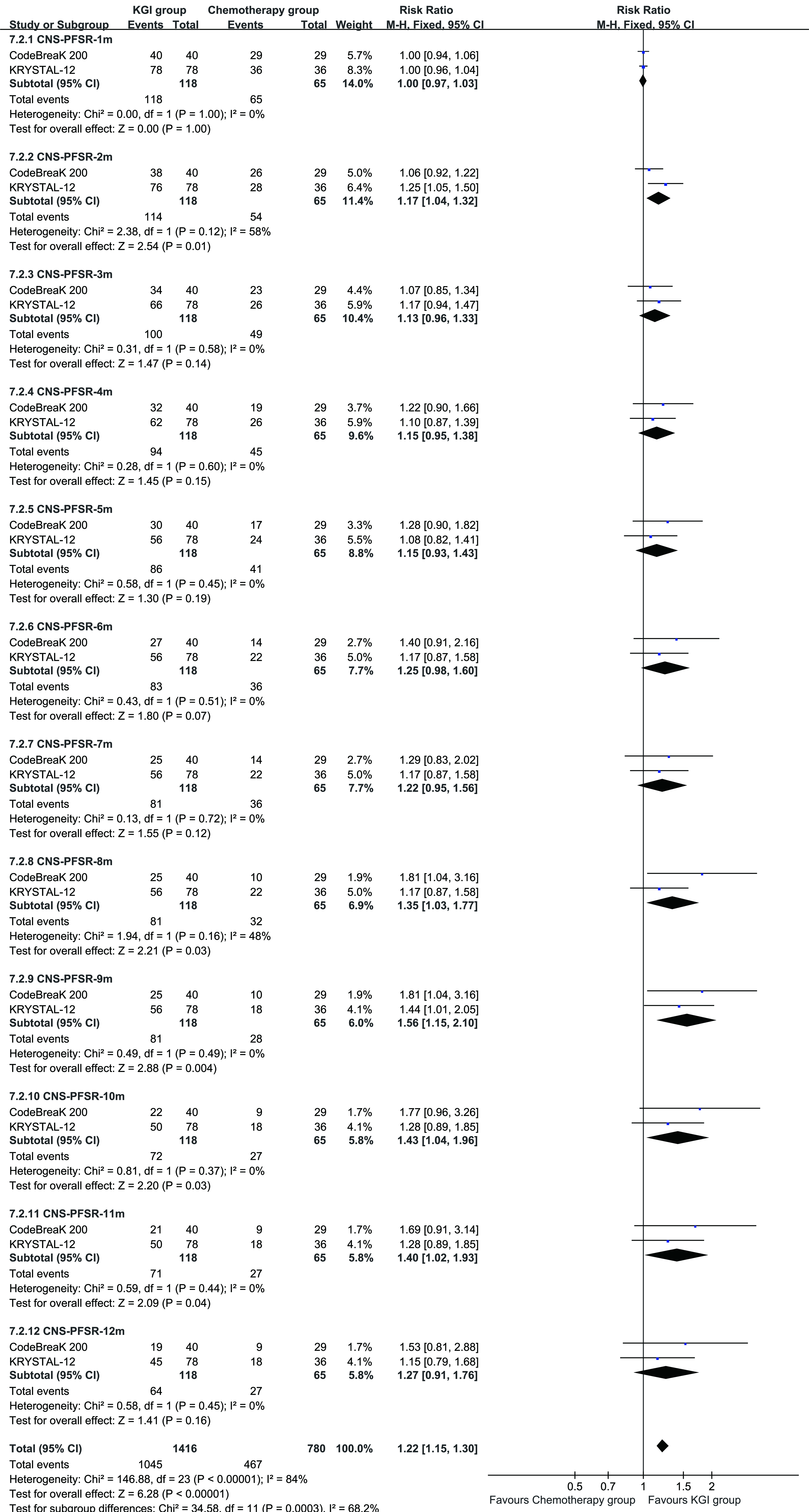

Supplement: Supplementary Figure S3 — Forest plots depicting CNS-PFSR at 1–12 months for KGIs compared with chemotherapy. [file Image3.tif]

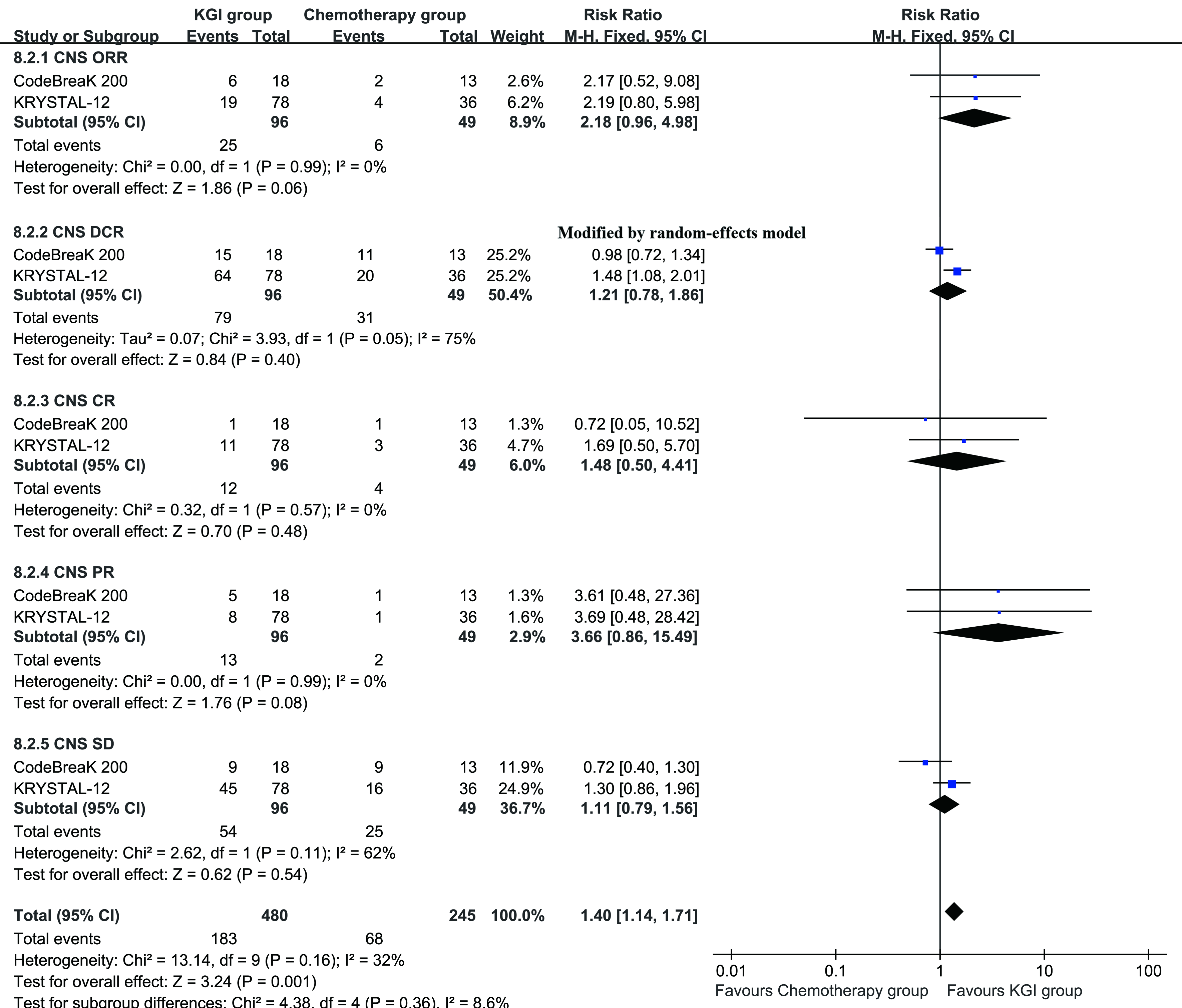

Supplement: Supplementary Figure S4 — Forest plots depicting CNS response rates for KGIs compared with chemotherapy. [file Image4.tif]

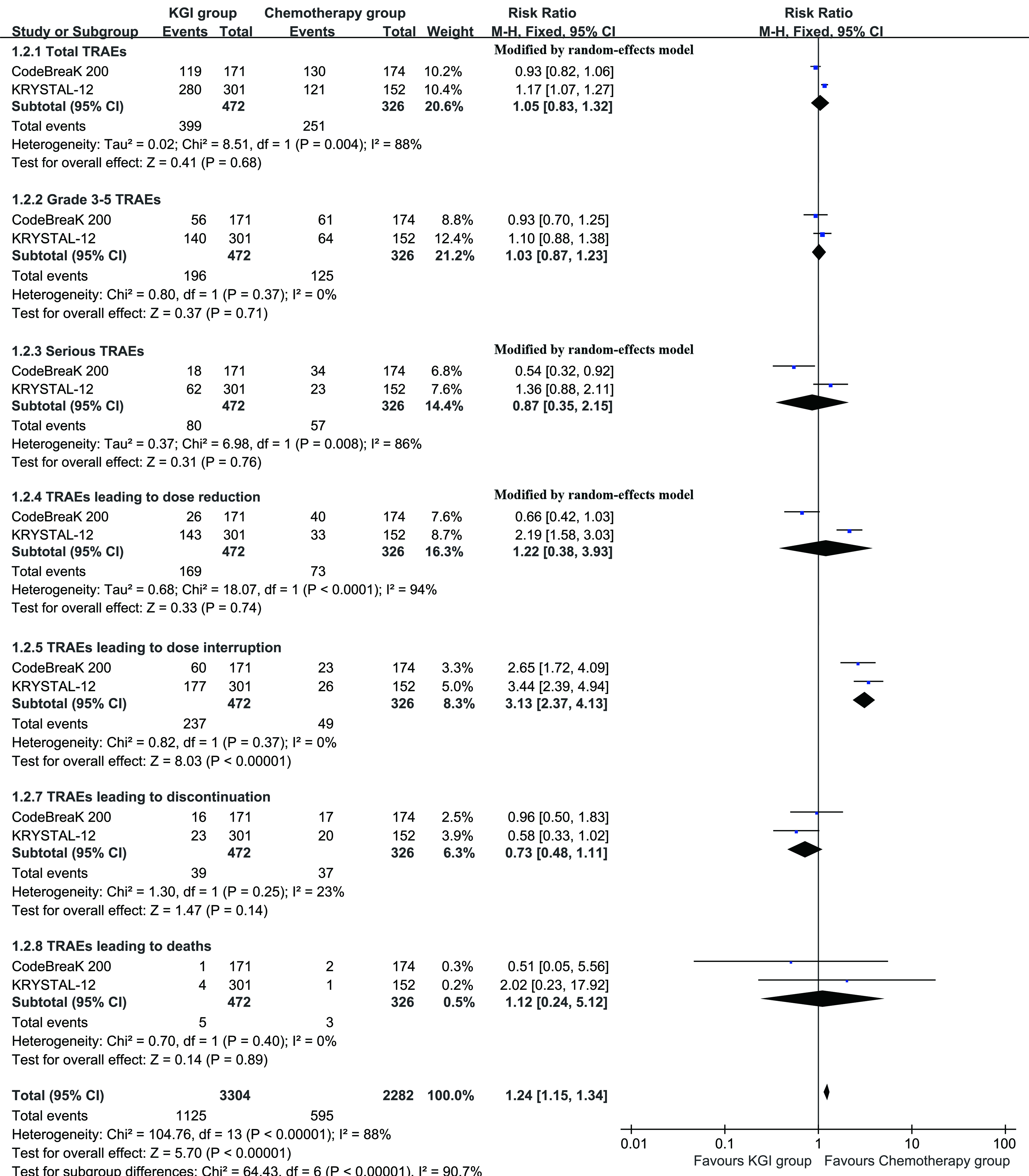

Supplement: Supplementary Figure S5 — Forest plots depicting safety summary for KGIs compared with chemotherapy. [file Image5.tif]
