# Supplementary material for: KRASG12C inhibitors versus chemotherapy alone for KRASG12C-mutated non-small cell lung cancer: a pooled analysis of CodeBreaK 200 and KRYSTAL-12 trials
Source: Front Oncol. 2026 Apr 15;16:1775677. doi: 10.3389/fonc.2026.1775677 (PMC13124570; doi:10.3389/fonc.2026.1775677)
Supplement: Supplementary Table S1 — Detailed literature search strategy. [file Table1.docx]

**Table S1** Detailed literature search strategy.

| **PubMed**  The database was searched on November 24, 2025, n=172.  Search Strategy:  **#1 Search: KRAS[Title/Abstract] OR K-RAS[Title/Abstract] Sort by: Most Recent n = 30115**  **#2 Lung cancer[Title/Abstract] OR NSCLC[Title/Abstract] OR Lung adenocarcinoma[Title/Abstract] OR Lung squamous cell carcinoma[Title/Abstract] Sort by: Most Recent n = 261563**  **#3 Randomized[Title/Abstract]) OR Randomly[Title/Abstract] OR Randomised[Title/Abstract] n = 1294434**  **#1 and #2 and #3 n = 172** |
| --- |
| **Web of Science**  The database was searched on November 24, 2025, n=188.  Search Strategy:  KRAS OR K-RAS (Abstract) AND Lung cancer OR NSCLC OR Lung adenocarcinoma OR Lung squamous cell carcinoma (Abstract) AND Randomized OR Randomly OR Randomised (Abstract) and Preprint Citation Index (Exclude - Database) |
| **EMBASE**  The database was searched on November 24, 2025, n=283.  Search Strategy:  (KRAS:ti,ab,kw OR K-RAS:ti,ab,kw) AND (Lung cancer:ti,ab,kw OR NSCLC:ti,ab,kw OR Lung adenocarcinoma:ti,ab,kw OR Lung squamous cell carcinoma:ti,ab,kw) AND **(Randomly**:ti,ab,kw **OR Randomised**:ti,ab,kw **OR Randomized**:ti,ab,kw**)** |
| **Cochrane Library**  The database was searched on November 24, 2025, n=51.  Search Strategy:  (KRAS OR K-RAS**)** in Title Abstract Keyword AND (Lung cancer OR NSCLC OR Lung adenocarcinoma OR Lung squamous cell carcinoma**)** in Title Abstract Keyword AND (**Randomized OR Randomly OR Randomised)** in Title Abstract Keyword - (Word variations have been searched) |
| **ScienceDirect**  The database was searched on November 24, 2025, n=922.  Search Strategy:  Title, abstract, keywords: ((“KRAS” OR “K-RAS”) AND (“Lung cancer” OR “NSCLC” OR “Lung adenocarcinoma” OR “Lung squamous cell carcinoma”) AND (“**Randomized**” **OR Randomly**” **OR** “**Randomised**”)) |
| **Scopus**  The database was searched on November 24, 2025, n=272.  Search Strategy:  (TITLE-ABS-KEY (KRAS OR K-RAS) AND TITLE-ABS-KEY (Lung cancer OR NSCLC OR Lung adenocarcinoma OR Lung squamous cell carcinoma) AND TITLE-ABS-KEY (Randomized OR Randomly OR Randomised)) |

**Note:** The combined text and medical subject heading (MeSH) terms used were: “**KRAS**”, “Lung cancer”, and “**Randomized**”.
