# Supplementary material for: KRASG12C inhibitors versus chemotherapy alone for KRASG12C-mutated non-small cell lung cancer: a pooled analysis of CodeBreaK 200 and KRYSTAL-12 trials
Source: Front Oncol. 2026 Apr 15;16:1775677. doi: 10.3389/fonc.2026.1775677 (PMC13124570; doi:10.3389/fonc.2026.1775677)
Supplement: Supplementary Table S2 — Methodological quality of included studies assessed by the Jadad scale. [file Table2.doc]

**Table S2** Methodological quality of included studies assessed by the Jadad scale.

| **Study** | | **Randomization** | **Concealment of allocation** | **Double blinding** | **Withdrawals and dropouts** | **Quality (score)** |
| --- | --- | --- | --- | --- | --- | --- |
| CodeBreaK 200 (NCT04303780) | Dingemans 2025[15], Waterhouse 2024[16], de Langen 2023[10] | ** | ** | * | * | 7 |
| KRYSTAL-12 (NCT04685135) | Barlesi 2025[11] | ** | ** | * | * | 7 |
