# Supplementary material for: KRASG12C inhibitors versus chemotherapy alone for KRASG12C-mutated non-small cell lung cancer: a pooled analysis of CodeBreaK 200 and KRYSTAL-12 trials
Source: Front Oncol. 2026 Apr 15;16:1775677. doi: 10.3389/fonc.2026.1775677 (PMC13124570; doi:10.3389/fonc.2026.1775677)
Supplement: Supplementary Table S3 — GRADE evaluation of evidence by treatment modality and study design. [file Table3.doc]

**Table S3** GRADE evaluation of evidence by treatment modality and study design.

| **Primary outcomes** | **No. of Participants** | | **Differences (95%CI) ^a^** | **Quality Assessment** | | | | | **Quality** |
| --- | --- | --- | --- | --- | --- | --- | --- | --- | --- |
|  | **KGI** | **Chemotherapy** |  | **Risk of Bias ^b^** | **Inconsistency** | **Indirectness** | **Imprecision** | **Publication Bias ^c^** |  |
| **Survival** |  |  |  |  |  |  |  |  |  |
| PFS | 472 | 326 | 0.62 [0.51, 0.74] | Low | No inconsistency | No indirectness | No imprecision | Unlikely | High |
| CNS-PFS | 472 | 326 | 0.55 [0.32, 0.94] | Low | No inconsistency | No indirectness | No imprecision | Unlikely | High |
| **Survival rate** |  |  |  |  |  |  |  |  |  |
| **PFSR** |  |  |  |  |  |  |  |  |  |
| PFSR-1m | 460/472 | 304/326 | 1.05 [1.02, 1.08] | Low | No inconsistency | No indirectness | No imprecision | Unlikely | High |
| PFSR-2m | 400/472 | 219/326 | 1.28 [1.17, 1.39] | Low | No inconsistency | No indirectness | No imprecision | Unlikely | High |
| PFSR-3m | 335/472 | 178/326 | 1.31 [1.17, 1.48] | Low | No inconsistency | No indirectness | No imprecision | Unlikely | High |
| PFSR-4m | 297/472 | 160/326 | 1.30 [1.14, 1.49] | Low | No inconsistency | No indirectness | No imprecision | Unlikely | High |
| PFSR-5m | 249/472 | 144/326 | 1.21 [1.04, 1.41] | Low | No inconsistency | No indirectness | No imprecision | Unlikely | High |
| PFSR-6m | 212/472 | 113/326 | 1.32 [1.10, 1.59] | Low | No inconsistency | No indirectness | No imprecision | Unlikely | High |
| PFSR-7m | 190/472 | 92/326 | 1.49 [1.21, 1.84] | Low | No inconsistency | No indirectness | No imprecision | Unlikely | High |
| PFSR-8m | 182/472 | 80/326 | 1.62 [1.30, 2.03] | Low | No inconsistency | No indirectness | No imprecision | Unlikely | High |
| PFSR-9m | 154/472 | 59/326 | 1.86 [1.42, 2.44] | Low | No inconsistency | No indirectness | No imprecision | Unlikely | High |
| PFSR-10m | 143/472 | 39/326 | 2.65 [1.90, 3.71] | Low | No inconsistency | No indirectness | No imprecision | Unlikely | High |
| PFSR-11m | 124/472 | 36/326 | 2.46 [1.73, 3.51] | Low | No inconsistency | No indirectness | No imprecision | Unlikely | High |
| PFSR-12m | 110/472 | 31/326 | 2.51 [1.71, 3.66] | Low | No inconsistency | No indirectness | No imprecision | Unlikely | High |
| **CNS-PFSR** |  |  |  |  |  |  |  |  |  |
| CNS-PFSR-1m | 118/118 | 65/65 | 1.00 [0.97, 1.03] | Low | No inconsistency | No indirectness | No imprecision | Unlikely | High |
| CNS-PFSR-2m | 114/118 | 54/65 | 1.17 [1.04, 1.32] | Low | No inconsistency | No indirectness | No imprecision | Unlikely | High |
| CNS-PFSR-3m | 100/118 | 49/65 | 1.13 [0.96, 1.33] | Low | No inconsistency | No indirectness | No imprecision | Unlikely | High |
| CNS-PFSR-4m | 94/118 | 45/65 | 1.15 [0.95, 1.38] | Low | No inconsistency | No indirectness | No imprecision | Unlikely | High |
| CNS-PFSR-5m | 86/118 | 41/65 | 1.15 [0.93, 1.43] | Low | No inconsistency | No indirectness | No imprecision | Unlikely | High |
| CNS-PFSR-6m | 83/118 | 36/65 | 1.25 [0.98, 1.60] | Low | No inconsistency | No indirectness | No imprecision | Unlikely | High |
| CNS-PFSR-7m | 81/118 | 36/65 | 1.22 [0.95, 1.56] | Low | No inconsistency | No indirectness | No imprecision | Unlikely | High |
| CNS-PFSR-8m | 81/118 | 32/65 | 1.35 [1.03, 1.77] | Low | No inconsistency | No indirectness | No imprecision | Unlikely | High |
| CNS-PFSR-9m | 81/118 | 28/65 | 1.56 [1.15, 2.10] | Low | No inconsistency | No indirectness | No imprecision | Unlikely | High |
| CNS-PFSR-10m | 72/118 | 27/65 | 1.43 [1.04, 1.96] | Low | No inconsistency | No indirectness | No imprecision | Unlikely | High |
| CNS-PFSR-11m | 71/118 | 27/65 | 1.40 [1.02, 1.93] | Low | No inconsistency | No indirectness | No imprecision | Unlikely | High |
| CNS-PFSR-12m | 64/118 | 27/65 | 1.27 [0.91, 1.76] | Low | No inconsistency | No indirectness | No imprecision | Unlikely | High |
| **Subgroup analysis** |  |  |  |  |  |  |  |  |  |
| **PFS** |  |  |  |  |  |  |  |  |  |
| Total | 798 | | 0.62 [0.51, 0.74] | Low | No inconsistency | No indirectness | No imprecision | Unlikely | High |
| Age - < 65 years | 420 | | 0.62 [0.48, 0.79] | Low | No inconsistency | No indirectness | No imprecision | Unlikely | High |
| Age - > 65 years | 378 | | 0.62 [0.46, 0.83] | Low | No inconsistency | No indirectness | No imprecision | Unlikely | High |
| Sex - Female | 291 | | 0.67 [0.48, 0.93] | Low | No inconsistency | No indirectness | No imprecision | Unlikely | High |
| Sex - Male | 507 | | 0.55 [0.44, 0.70] | Low | No inconsistency | No indirectness | No imprecision | Unlikely | High |
| Geographic region - Asia | 161 | | 0.56 [0.36, 0.86] | Low | No inconsistency | No indirectness | No imprecision | Unlikely | High |
| Geographic region - Non-Asia | 635 | | 0.63 [0.51, 0.78] | Low | No inconsistency | No indirectness | No imprecision | Unlikely | High |
| ECOG PS - 0 | 261 | | 0.53 [0.37, 0.76] | Low | No inconsistency | No indirectness | No imprecision | Unlikely | High |
| ECOG PS - 1 | 536 | | 0.61 [0.49, 0.76] | Low | No inconsistency | No indirectness | No imprecision | Unlikely | High |
| Smoking status - Current/former | 758 | | 0.61 [0.50, 0.75] | Low | No inconsistency | No indirectness | No imprecision | Unlikely | High |
| Smoking status - Never | 26 | | 0.70 [0.26, 1.88] | Low | No inconsistency | No indirectness | No imprecision | Unlikely | High |
| Brain metastases - Yes | 232 | | 0.60 [0.43, 0.83] | Low | No inconsistency | No indirectness | No imprecision | Unlikely | High |
| Brain metastases - No | 566 | | 0.62 [0.50, 0.78] | Low | No inconsistency | No indirectness | No imprecision | Unlikely | High |
| Liver metastases - Yes | 129 | | 0.45 [0.29, 0.70] | Low | No inconsistency | No indirectness | No imprecision | Unlikely | High |
| Liver metastases - No | 669 | | 0.62 [0.51, 0.77] | Low | No inconsistency | No indirectness | No imprecision | Unlikely | High |
| Bone metastases - Yes | 257 | | 0.61 [0.45, 0.82] | Low | No inconsistency | No indirectness | No imprecision | Unlikely | High |
| Bone metastases - No | 541 | | 0.58 [0.46, 0.74] | Low | No inconsistency | No indirectness | No imprecision | Unlikely | High |
| PD-L1 expression - <1% | 207 | | 0.56 [0.39, 0.81] | Low | No inconsistency | No indirectness | No imprecision | Unlikely | High |
| PD-L1 expression - 1%-49% | 311 | | 0.58 [0.43, 0.78] | Low | No inconsistency | No indirectness | No imprecision | Unlikely | High |
| PD-L1 expression - >50% | 200 | | 0.68 [0.46, 1.01] | Low | No inconsistency | No indirectness | No imprecision | Unlikely | High |
| **Responses** |  |  |  |  |  |  |  |  |  |
| **Overall response** |  |  |  |  |  |  |  |  |  |
| ORR | 144/472 | 37/326 | 2.73 [1.93, 3.85] | Low | No inconsistency | No indirectness | No imprecision | Unlikely | High |
| DCR | 377/472 | 194/326 | 1.35 [1.22, 1.50] | Low | No inconsistency | No indirectness | No imprecision | Unlikely | High |
| CR | 5/472 | 0/326 | 4.21 [0.51, 34.63] | Low | No inconsistency | No indirectness | No imprecision | Unlikely | High |
| PR | 139/472 | 37/326 | 2.63 [1.86, 3.72] | Low | No inconsistency | No indirectness | No imprecision | Unlikely | High |
| SD | 233/472 | 157/326 | 1.04 [0.90, 1.20] | Low | No inconsistency | No indirectness | No imprecision | Unlikely | High |
| PD | 95/472 | 132/326 | 0.49 [0.39, 0.61] | Low | No inconsistency | No indirectness | No imprecision | Unlikely | High |
| **CNS response** |  |  |  |  |  |  |  |  |  |
| CNS-ORR | 25/96 | 6/49 | 2.18 [0.96, 4.98] | Low | No inconsistency | No indirectness | No imprecision | Unlikely | High |
| CNS-DCR | 79/96 | 31/49 | 1.21 [0.78, 1.86] | Low | Serious (-1) | No indirectness | No imprecision | Unlikely | Medium |
| CNS-CR | 12/96 | 4/49 | 1.48 [0.50, 4.41] | Low | No inconsistency | No indirectness | No imprecision | Unlikely | High |
| CNS-PR | 13/96 | 2/49 | 3.66 [0.86, 15.49] | Low | No inconsistency | No indirectness | No imprecision | Unlikely | High |
| CNS-SD | 54/96 | 25/49 | 1.11 [0.79, 1.56] | Low | No inconsistency | No indirectness | No imprecision | Unlikely | High |
| CNS-PD | 17/96 | 18/49 | 0.47 [0.27, 0.82] | Low | No inconsistency | No indirectness | No imprecision | Unlikely | High |
| **Safety summary** |  |  |  |  |  |  |  |  |  |
| Total TRAEs | 399/472 | 251/326 | 1.05 [0.83, 1.32] | Low | Serious (-1) | No indirectness | No imprecision | Unlikely | Medium |
| Grade 3-5 TRAEs | 196/472 | 125/326 | 1.03 [0.87, 1.23] | Low | No inconsistency | No indirectness | No imprecision | Unlikely | High |
| Serious TRAEs | 80/472 | 57/326 | 0.87 [0.35, 2.15] | Low | Serious (-1) | No indirectness | No imprecision | Unlikely | Medium |
| TRAEs leading to dose reduction | 169/472 | 73/326 | 1.22 [0.38, 3.93] | Low | Serious (-1) | No indirectness | No imprecision | Unlikely | Medium |
| TRAEs leading to dose interruption | 237/472 | 49/326 | 3.13 [2.37, 4.13] | Low | No inconsistency | No indirectness | No imprecision | Unlikely | High |
| TRAEs leading to discontinuation | 39/472 | 37/326 | 0.73 [0.45, 1.18] | Low | Serious (-1) | No indirectness | No imprecision | Unlikely | Medium |
| TRAEs leading to deaths | 5/472 | 3/326 | 1.12 [0.24, 5.12] | Low | No inconsistency | No indirectness | No imprecision | Unlikely | High |
| **TRAEs** |  |  |  |  |  |  |  |  |  |
| **All grade** |  |  |  |  |  |  |  |  |  |
| Diarrhea | 215/472 | 71/326 | 1.93 [1.54, 2.42] | Low | No inconsistency | No indirectness | No imprecision | Unlikely | High |
| Nausea | 125/472 | 57/326 | 1.26 [0.55, 2.87] | Low | Serious (-1) | No indirectness | No imprecision | Unlikely | Medium |
| Vomiting | 111/472 | 19/326 | 2.22 [0.32, 15.71] | Low | Serious (-1) | No indirectness | No imprecision | Unlikely | Medium |
| AST increased | 109/472 | 0/326 | 68.88 [8.94, 530.86] | Low | No inconsistency | No indirectness | No imprecision | Unlikely | High |
| ALT increased | 107/472 | 4/326 | 13.43 [5.39, 33.49] | Low | No inconsistency | No indirectness | No imprecision | Unlikely | High |
| Blood creatinine increased | 59/301 | 2/152 | 14.90 [3.69, 60.15] | Low | No inconsistency | No indirectness | No imprecision | Unlikely | High |
| Decreased appetite | 88/472 | 51/326 | 1.07 [0.78, 1.48] | Low | No inconsistency | No indirectness | No imprecision | Unlikely | High |
| Asthenia | 63/472 | 54/326 | 0.67 [0.48, 0.94] | Low | No inconsistency | No indirectness | No imprecision | Unlikely | High |
| Anemia | 62/472 | 69/326 | 0.39 [0.11, 1.41] | Low | Serious (-1) | No indirectness | No imprecision | Unlikely | Medium |
| γ-Glutamyltransferase increased | 38/301 | 3/152 | 6.40 [2.01, 20.39] | Low | No inconsistency | No indirectness | No imprecision | Unlikely | High |
| Fatigue | 58/472 | 58/326 | 0.60 [0.15, 2.37] | Low | Serious (-1) | No indirectness | No imprecision | Unlikely | Medium |
| Blood ALP increased | 55/472 | 2/326 | 17.51 [4.11, 74.67] | Low | No inconsistency | No indirectness | No imprecision | Unlikely | High |
| Lipase increased | 35/301 | 2/152 | 8.84 [2.15, 36.25] | Low | No inconsistency | No indirectness | No imprecision | Unlikely | High |
| Abdominal pain | 9/171 | 6/174 | 1.53 [0.56, 4.20] | Low | No inconsistency | No indirectness | No imprecision | Unlikely | High |
| White blood cell count decreased | 10/301 | 14/152 | 0.36 [0.16, 0.79] | Low | No inconsistency | No indirectness | No imprecision | Unlikely | High |
| Neutrophil count decreased | 9/301 | 23/152 | 0.20 [0.09, 0.42] | Low | No inconsistency | No indirectness | No imprecision | Unlikely | High |
| Stomatitis | 14/472 | 31/326 | 0.20 [0.02, 1.77] | Low | Serious (-1) | No indirectness | No imprecision | Unlikely | Medium |
| Constipation | 5/171 | 16/174 | 0.32 [0.12, 0.85] | Low | No inconsistency | No indirectness | No imprecision | Unlikely | High |
| Dysgeusia | 4/171 | 13/174 | 0.31 [0.10, 0.94] | Low | No inconsistency | No indirectness | No imprecision | Unlikely | High |
| Neutropenia | 10/472 | 37/326 | 0.17 [0.08, 0.36] | Low | No inconsistency | No indirectness | No imprecision | Unlikely | High |
| Myalgia | 3/171 | 13/174 | 0.23 [0.07, 0.81] | Low | No inconsistency | No indirectness | No imprecision | Unlikely | High |
| Arthralgia | 2/171 | 10/174 | 0.20 [0.05, 0.92] | Low | No inconsistency | No indirectness | No imprecision | Unlikely | High |
| Malaise | 2/171 | 9/174 | 0.23 [0.05, 1.03] | Low | No inconsistency | No indirectness | No imprecision | Unlikely | High |
| Alopecia | 4/472 | 65/326 | 0.04 [0.02, 0.12] | Low | No inconsistency | No indirectness | No imprecision | Unlikely | High |
| Mucositis | 1/171 | 10/174 | 0.10 [0.01, 0.79] | Low | No inconsistency | No indirectness | No imprecision | Unlikely | High |
| Pyrexia | 1/171 | 8/174 | 0.13 [0.02, 1.01] | Low | No inconsistency | No indirectness | No imprecision | Unlikely | High |
| Neuropathy peripheral | 0/171 | 15/174 | 0.03 [0.00, 0.54] | Low | No inconsistency | No indirectness | No imprecision | Unlikely | High |
| Oedema peripheral | 0/171 | 14/174 | 0.04 [0.00, 0.58] | Low | No inconsistency | No indirectness | No imprecision | Unlikely | High |
| Febrile neutropenia | 0/171 | 8/174 | 0.06 [0.00, 1.03] | Low | No inconsistency | No indirectness | No imprecision | Unlikely | High |
| Pneumonia | 0/171 | 7/174 | 0.07 [0.00, 1.18] | Low | No inconsistency | No indirectness | No imprecision | Unlikely | High |
| **Grade 3-5** |  |  |  |  |  |  |  |  |  |
| Diarrhea | 36/472 | 9/326 | 2.89 [0.58, 14.41] | Low | Serious (-1) | No indirectness | No imprecision | Unlikely | Medium |
| ALT increased | 36/472 | 0/326 | 25.38 [3.46, 186.06] | Low | No inconsistency | No indirectness | No imprecision | Unlikely | High |
| AST increased | 28/472 | 0/326 | 19.58 [2.62, 146.31] | Low | No inconsistency | No indirectness | No imprecision | Unlikely | High |
| γ-Glutamyltransferase increased | 15/301 | 0/152 | 15.71 [0.95, 260.72] | Low | No inconsistency | No indirectness | No imprecision | Unlikely | High |
| Lipase increased | 12/301 | 0/152 | 12.67 [0.75, 212.49] | Low | No inconsistency | No indirectness | No imprecision | Unlikely | High |
| Asthenia | 14/472 | 18/326 | 0.43 [0.22, 0.86] | Low | No inconsistency | No indirectness | No imprecision | Unlikely | High |
| Fatigue | 11/472 | 12/326 | 0.49 [0.03, 7.37] | Low | Serious (-1) | No indirectness | No imprecision | Unlikely | Medium |
| Nausea | 11/472 | 2/326 | 3.47 [0.73, 16.50] | Low | No inconsistency | No indirectness | No imprecision | Unlikely | High |
| Anemia | 11/472 | 11/326 | 0.60 [0.25, 1.43] | Low | No inconsistency | No indirectness | No imprecision | Unlikely | High |
| Blood ALP increased | 10/472 | 0/326 | 7.98 [1.07, 59.66] | Low | No inconsistency | No indirectness | No imprecision | Unlikely | High |
| Decreased appetite | 7/472 | 2/326 | 1.97 [0.51, 7.58] | Low | No inconsistency | No indirectness | No imprecision | Unlikely | High |
| Neutrophil count decreased | 4/301 | 16/152 | 0.13 [0.04, 0.37] | Low | No inconsistency | No indirectness | No imprecision | Unlikely | High |
| Abdominal pain | 2/171 | 0/174 | 5.09 [0.25, 105.19] | Low | No inconsistency | No indirectness | No imprecision | Unlikely | High |
| Vomiting | 5/472 | 1/326 | 2.52 [0.30, 21.42] | Low | No inconsistency | No indirectness | No imprecision | Unlikely | High |
| Neutropenia | 5/472 | 32/326 | 0.10 [0.04, 0.28] | Low | No inconsistency | No indirectness | No imprecision | Unlikely | High |
| White blood cell count decreased | 2/301 | 7/152 | 0.14 [0.03, 0.69] | Low | No inconsistency | No indirectness | No imprecision | Unlikely | High |
| Malaise | 1/171 | 1/174 | 1.02 [0.06, 16.14] | Low | No inconsistency | No indirectness | No imprecision | Unlikely | High |
| Blood creatinine increased | 1/301 | 0/152 | 1.52 [0.06, 37.09] | Low | No inconsistency | No indirectness | No imprecision | Unlikely | High |
| Stomatitis | 0/472 | 4/326 | 0.15 [0.02, 1.20] | Low | No inconsistency | No indirectness | No imprecision | Unlikely | High |
| Neuropathy peripheral | 0/171 | 1/174 | 0.34 [0.01, 8.27] | Low | No inconsistency | No indirectness | No imprecision | Unlikely | High |
| Oedema peripheral | 0/171 | 1/174 | 0.34 [0.01, 8.27] | Low | No inconsistency | No indirectness | No imprecision | Unlikely | High |
| Myalgia | 0/171 | 2/174 | 0.20 [0.01, 4.21] | Low | No inconsistency | No indirectness | No imprecision | Unlikely | High |
| Arthralgia | 0/171 | 1/174 | 0.34 [0.01, 8.27] | Low | No inconsistency | No indirectness | No imprecision | Unlikely | High |
| Mucositis | 0/171 | 2/174 | 0.20 [0.01, 4.21] | Low | No inconsistency | No indirectness | No imprecision | Unlikely | High |
| Febrile neutropenia | 0/171 | 8/174 | 0.06 [0.00, 1.03] | Low | No inconsistency | No indirectness | No imprecision | Unlikely | High |
| Pneumonia | 0/171 | 5/174 | 0.09 [0.01, 1.66] | Low | No inconsistency | No indirectness | No imprecision | Unlikely | High |

**Abbreviations:** AE: Adverse event; ALP: Alkaline phosphatase; ALT: Alanine aminotransferase; AST: Aspartate aminotransferase; CI: Confidence interval; CNS: Central nervous system; CR: Complete response; DCR: Disease control rate; ECOG PS: Eastern cooperative oncology group performance status; GRADE: Grading of recommendations assessment, development, and evaluation; HR: Hazard ratio; *I²*: I-squared statistic; KGI: KRAS^G12C^ inhibitor; KRAS: Kirsten rat sarcoma viral oncogene homolog; M/F: Male/Female; NSCLC: Non-small cell lung cancer; ORR: Objective response rate; OS: Overall survival; P: Probability; PD: Progressive disease; PD-L1: Programmed death-ligand 1; PFS: Progression-free survival; PFSR: Progression-free survival rate; PR: Partial response; RCT: Randomized controlled trial; RR: Risk ratio; SD: Stable disease; TRAEs: Treatment-related adverse events.

^a^ Differences: HR for PFS and CNS-PFS; RR for PFSR, CNS-PFSR, responses, and TRAEs.

^b^ Risk of bias assessed using the Jadad scale for randomized controlled trials.

^c^ Publication bias was explored through visual inspection of the funnel plots.
