# Supplementary material for: KRASG12C inhibitors versus chemotherapy alone for KRASG12C-mutated non-small cell lung cancer: a pooled analysis of CodeBreaK 200 and KRYSTAL-12 trials
Source: Front Oncol. 2026 Apr 15;16:1775677. doi: 10.3389/fonc.2026.1775677 (PMC13124570; doi:10.3389/fonc.2026.1775677)
Supplement: Supplementary Table S4 — Any grade treatment-related adverse events. [file Table4.doc]

**Table S4** Any grade treatment-related adverse events.

| **TRAEs** | **KGI** | |  | **Chemotherapy** | | **Risk ratio [95% CI]** | **P** |
| --- | --- | --- | --- | --- | --- | --- | --- |
|  | **Event/total** | **%** |  | **Event/total** | **%** |  |  |
| Diarrhea | 215/472 | 45.55% |  | 71/326 | 21.78% | 1.93 [1.54, 2.42] | < 0.00001 |
| Nausea | 125/472 | 26.48% |  | 57/326 | 17.48% | 1.26 [0.55, 2.87] | 0.58 |
| Vomiting | 111/472 | 23.52% |  | 19/326 | 5.83% | 2.22 [0.32, 15.71] | 0.42 |
| AST increased | 109/472 | 23.09% |  | 0/326 | 0.00% | 68.88 [8.94, 530.86] | < 0.0001 |
| ALT increased | 107/472 | 22.67% |  | 4/326 | 1.23% | 13.43 [5.39, 33.49] | < 0.00001 |
| Blood creatinine increased | 59/301 | 19.60% |  | 2/152 | 1.32% | 14.90 [3.69, 60.15] | 0.0001 |
| Decreased appetite | 88/472 | 18.64% |  | 51/326 | 15.64% | 1.07 [0.78, 1.48] | 0.66 |
| Asthenia | 63/472 | 13.35% |  | 54/326 | 16.56% | 0.67 [0.48, 0.94] | 0.02 |
| Anemia | 62/472 | 13.14% |  | 69/326 | 21.17% | 0.39 [0.11, 1.41] | 0.15 |
| γ-Glutamyltransferase increased | 38/301 | 12.62% |  | 3/152 | 1.97% | 6.40 [2.01, 20.39] | 0.002 |
| Fatigue | 58/472 | 12.29% |  | 58/326 | 17.79% | 0.60 [0.15, 2.37] | 0.47 |
| Blood ALP increased | 55/472 | 11.65% |  | 2/326 | 0.61% | 17.51 [4.11, 74.67] | 0.0001 |
| Lipase increased | 35/301 | 11.63% |  | 2/152 | 1.32% | 8.84 [2.15, 36.25] | 0.002 |
| Abdominal pain | 9/171 | 5.26% |  | 6/174 | 3.45% | 1.53 [0.56, 4.20] | 0.41 |
| White blood cell count decreased | 10/301 | 3.32% |  | 14/152 | 9.21% | 0.36 [0.16, 0.79] | 0.01 |
| Neutrophil count decreased | 9/301 | 2.99% |  | 23/152 | 15.13% | 0.20 [0.09, 0.42] | < 0.0001 |
| Stomatitis | 14/472 | 2.97% |  | 31/326 | 9.51% | 0.20 [0.02, 1.77] | 0.15 |
| Constipation | 5/171 | 2.92% |  | 16/174 | 9.20% | 0.32 [0.12, 0.85] | 0.02 |
| Dysgeusia | 4/171 | 2.34% |  | 13/174 | 7.47% | 0.31 [0.10, 0.94] | 0.04 |
| Neutropenia | 10/472 | 2.12% |  | 37/326 | 11.35% | 0.17 [0.08, 0.36] | < 0.00001 |
| Myalgia | 3/171 | 1.75% |  | 13/174 | 7.47% | 0.23 [0.07, 0.81] | 0.02 |
| Arthralgia | 2/171 | 1.17% |  | 10/174 | 5.75% | 0.20 [0.05, 0.92] | 0.04 |
| Malaise | 2/171 | 1.17% |  | 9/174 | 5.17% | 0.23 [0.05, 1.03] | 0.05 |
| Alopecia | 4/472 | 0.85% |  | 65/326 | 19.94% | 0.04 [0.02, 0.12] | < 0.00001 |
| Mucositis | 1/171 | 0.58% |  | 10/174 | 5.75% | 0.10 [0.01, 0.79] | 0.03 |
| Pyrexia | 1/171 | 0.58% |  | 8/174 | 4.60% | 0.13 [0.02, 1.01] | 0.05 |
| Neuropathy peripheral | 0/171 | 0.00% |  | 15/174 | 8.62% | 0.03 [0.00, 0.54] | 0.02 |
| Oedema peripheral | 0/171 | 0.00% |  | 14/174 | 8.05% | 0.04 [0.00, 0.58] | 0.02 |
| Febrile neutropenia | 0/171 | 0.00% |  | 8/174 | 4.60% | 0.06 [0.00, 1.03] | 0.05 |
| Pneumonia | 0/171 | 0.00% |  | 7/174 | 4.02% | 0.07 [0.00, 1.18] | 0.06 |

**Abbreviations:** ALP: Alkaline phosphatase; ALT: Alanine aminotransferase; AST: Aspartate aminotransferase; CI: Confidence interval; CI: Confidence interval; *I²*: I-squared statistic; KGI: KRAS^G12C^ inhibitor; P: Probability; RR: Risk ratio; TRAE: Treatment-related adverse event.
