# Supplementary material for: KRASG12C inhibitors versus chemotherapy alone for KRASG12C-mutated non-small cell lung cancer: a pooled analysis of CodeBreaK 200 and KRYSTAL-12 trials
Source: Front Oncol. 2026 Apr 15;16:1775677. doi: 10.3389/fonc.2026.1775677 (PMC13124570; doi:10.3389/fonc.2026.1775677)
Supplement: Supplementary Table S5 — Grade 3–5 treatment-related adverse events. [file Table5.doc]

**Table S5** Grade 3-5 treatment-related adverse events.

| **TRAEs** | **KGI** | |  | **Chemotherapy** | | **Risk ratio [95% CI]** | **P** |
| --- | --- | --- | --- | --- | --- | --- | --- |
|  | **Event/total** | **%** |  | **Event/total** | **%** |  |  |
| Diarrhea | 36/472 | 7.63% |  | 9/326 | 2.76% | 2.89 [0.58, 14.41] | 0.20 |
| ALT increased | 36/472 | 7.63% |  | 0/326 | 0.00% | 25.38 [3.46, 186.06] | 0.001 |
| AST increased | 28/472 | 5.93% |  | 0/326 | 0.00% | 19.58 [2.62, 146.31] | 0.004 |
| γ-Glutamyltransferase increased | 15/301 | 4.98% |  | 0/152 | 0.00% | 15.71 [0.95, 260.72] | 0.05 |
| Lipase increased | 12/301 | 3.99% |  | 0/152 | 0.00% | 12.67 [0.75, 212.49] | 0.08 |
| Asthenia | 14/472 | 2.97% |  | 18/326 | 5.52% | 0.43 [0.22, 0.86] | 0.02 |
| Fatigue | 11/472 | 2.33% |  | 12/326 | 3.68% | 0.49 [0.03, 7.37] | 0.61 |
| Nausea | 11/472 | 2.33% |  | 2/326 | 0.61% | 3.47 [0.73, 16.50] | 0.12 |
| Anemia | 11/472 | 2.33% |  | 11/326 | 3.37% | 0.60 [0.25, 1.43] | 0.25 |
| Blood ALP increased | 10/472 | 2.12% |  | 0/326 | 0.00% | 7.98 [1.07, 59.66] | 0.04 |
| Decreased appetite | 7/472 | 1.48% |  | 2/326 | 0.61% | 1.97 [0.51, 7.58] | 0.32 |
| Neutrophil count decreased | 4/301 | 1.33% |  | 16/152 | 10.53% | 0.13 [0.04, 0.37] | 0.0002 |
| Abdominal pain | 2/171 | 1.17% |  | 0/174 | 0.00% | 5.09 [0.25, 105.19] | 0.29 |
| Vomiting | 5/472 | 1.06% |  | 1/326 | 0.31% | 2.52 [0.30, 21.42] | 0.40 |
| Neutropenia | 5/472 | 1.06% |  | 32/326 | 9.82% | 0.10 [0.04, 0.28] | < 0.00001 |
| White blood cell count decreased | 2/301 | 0.66% |  | 7/152 | 4.61% | 0.14 [0.03, 0.69] | 0.01 |
| Malaise | 1/171 | 0.58% |  | 1/174 | 0.57% | 1.02 [0.06, 16.14] | 0.99 |
| Blood creatinine increased | 1/301 | 0.33% |  | 0/152 | 0.00% | 1.52 [0.06, 37.09] | 0.80 |
| Stomatitis | 0/472 | 0.00% |  | 4/326 | 1.23% | 0.15 [0.02, 1.20] | 0.07 |
| Neuropathy peripheral | 0/171 | 0.00% |  | 1/174 | 0.57% | 0.34 [0.01, 8.27] | 0.51 |
| Oedema peripheral | 0/171 | 0.00% |  | 1/174 | 0.57% | 0.34 [0.01, 8.27] | 0.51 |
| Myalgia | 0/171 | 0.00% |  | 2/174 | 1.15% | 0.20 [0.01, 4.21] | 0.30 |
| Arthralgia | 0/171 | 0.00% |  | 1/174 | 0.57% | 0.34 [0.01, 8.27] | 0.51 |
| Mucositis | 0/171 | 0.00% |  | 2/174 | 1.15% | 0.20 [0.01, 4.21] | 0.30 |
| Febrile neutropenia | 0/171 | 0.00% |  | 8/174 | 4.60% | 0.06 [0.00, 1.03] | 0.05 |
| Pneumonia | 0/171 | 0.00% |  | 5/174 | 2.87% | 0.09 [0.01, 1.66] | 0.11 |

**Abbreviations:** ALP: Alkaline phosphatase; ALT: Alanine aminotransferase; AST: Aspartate aminotransferase; CI: Confidence interval; *I²*: I-squared statistic; KGI: KRAS^G12C^ inhibitor; P: Probability; RR: Risk ratio; TRAE: Treatment-related adverse event.
